# Supplementary material for: Identification of Francisella tularensis Subspecies in a Clinical Setting Using MALDI-TOF MS: An In-House Francisella Library and Biomarkers
Source: Microorganisms. 2023 Mar 30;11(4):905. doi: 10.3390/microorganisms11040905 (PMC10146885; doi:10.3390/microorganisms11040905)
Supplement: Supplementary file 1 [file microorganisms-11-00905-s001.zip › Table S3 Selected biomarker masses matched with genes of possible proteins-proof.pdf]

Supplementary Table S3. Selected biomarker masses matched with genes of possible proteins

Selected biomarker (m/z value)	Matching protein[a]	Gene code	Strain	Accession number Protein protein / genome[b]	Charges (z-values)	theoretical posttranslational corrections
2195	50S ribosomal protein L36	50S-L36	FSC 147	ACD31328 / CP00915	+2	none
3101	50S ribosomal protein L33	50S-L33	TOBA-04	AFJ42863 / CP003402	+2	none
3128	Hypothetical protein	HypProt	FSC 774	HypProt[cb] / CP053850		Plus methyl
3679	Coldshock DNA-binding proteins	ColdShockP	TOBA-04	AFJ43499 / CP003402	+2	none
				AFJ43802/ CP003402		
3880-3881	50S ribosomal protein L29	50S-L29	CCUG 19701	ABZ86979 / CP000937	+2	none
4040	Unknown mass	UNK	Ft27	unkown		none
4387	BolA-like protein	BolA	CCUG 60119	AJC48275 / CP010427	+2	none
4455	50S ribosomal protein L27	50S-L27	LVS	AJI58752 / CP009694	+2	minus start Methionine
4477	50S ribosomal protein L27	50S-L27	TOBA-04	AFJ43684 / CP003402	+2	minus start Methionine
5182-5185 [Li et al, [Seibold et al]	50S ribosomal protein L34	50S-L34	Schu S4	CAG44869 / AJ74994		none
7759-77601	50S ribosomal protein L29	50S-L29	CCUG 19701	ABZ86979 / CP000937		none
8079	DNA-directed RNA polymerase Ω subunit	RNApolymΩ	U112	CAG45336 / AJ749949		minus start Methionine plus dimethyl
8522	phosphatase PAP2 family protein	PAP2	DSM 22475	APD49887 / CP018093	+3	plus trimethyl (start AA=L)
8923-8925	50S ribosomal protein L27	50S-L27	CCUG 19701	ABZ86366 / CP000937		minus start Methionine
9380	Histon-like protein form Beta	HU-beta	FSC 774	QOG55061 / CP053850		none
9394-9396	Histon-like protein form Beta	HU-beta	CCUG 19701	ABZ97761 / CP000937		none
9449	Histon-like protein form Beta	HU-beta	Schu S4	AJI60850 / CP009633		none
9476-9477	Histon-like protein form Beta	HU-beta	U112	CAG45260 / AJ749949		none
10215	Chaperonin protein, groES	GroES	FSC 774	QOG55434 / CP053850		none
	Parvulin-like peptidylprolyl isomerase	PPisom	FSC 774	QOG54986 / CP053850		none

BolA = bovine lymphocyte antigen; PAP Phosphatic acid phosphatase

[a]Matching gene determined with Tagident or Uniprot

[b]The protein and genome accessions are from the strains from which the biomarkers were selected

The genome sequence of each gene was translated to amino acid (AA) sequence to determine the mass, not the accession AA sequence

[c]sequence 391813..39188 complement CP053850 (almost identical to Hypothetical protein QEN42979, CP022952 *F. orientalis* strain FN0364)
